# Training of Tube Thoracostomy on Soft-Embalmed Cadavers According to Thiel and Dodge: What Do Experts Say?

**DOI:** 10.1016/j.atssr.2025.07.009

**Published:** 2025-07-31

**Authors:** Dariya Jaeger, Eric Hinrichs, Volkan Kösek, Burkhard Thiel, Ludger Hillejan, Morris Beshay, Ralf Schoppe, Sven Schumann, Gebhard Reiss, Georg Feigl, Bassam Redwan

**Affiliations:** 1 Institute of Anatomy and Clinical Morphology, University of Witten/Herdecke, Witten, Germany; 2 Department of Thoracic Surgery, Knappschaft Westfalen Hospital GmbH, Lünen, Germany; 3 Department of Thoracic Surgery, St. Raphael Ostercappeln Hospital, Osnabrück, Germany; 4 Department of Thoracic Surgery, University Hospital OWL, Bielefeld, Germany; 5 MoViDo gGmbH, Essen, Germany; 6 Institute of Anatomy, University Medical Center of the Johannes Gutenberg-University Mainz, Mainz, Germany

## Abstract

**Background:**

Tube thoracostomy is one of the most important invasive procedures performed in many medical specialties for various indications.

**Methods:**

A total of 13 experienced thoracic surgeons were asked to perform a single tube thoracostomy as a Bülau drain on a soft-embalmed human cadaver. The surgeons used a structured questionnaire with a modified Likert scale to rate the closeness to reality of the performed training on soft-preserved cadavers compared with standard performance on living patients. Two forms of soft embalming were available: Thiel’s, in 2 cadavers; and Dodge’s, in 2 cadavers. Six surgeons performed the procedure on Thiel embalmed cadavers, and the other 7 surgeons performed the procedure on Dodge embalmed cadavers.

**Results:**

The evaluation of the results showed a high degree of closeness to reality and a 100% recommendation rate of the training for both forms of preservation. No significant differences could be found between Dodge embalmed cadavers and Thiel embalmed cadavers.

**Conclusions:**

The use of soft-embalmed cadavers was rated very positively by experienced thoracic surgeons while performing tube thoracostomy. The evaluation results showed a high degree of realism of both embalming methods compared with performing the procedure on live patients. Further studies are needed to perform a competitive statistical analysis comparing Dodge embalmed cadavers and Thiel embalmed cadavers.

**In Short**

▪ Thiel’s and Dodge’s soft-embalming methods provide a realistic training modality for tube thoracostomy.
▪ A total of 13 experienced thoracic surgeons performed tube thoracostomies on human cadavers that had been embalmed using Thiel’s and Dodge’s soft-embalming methods. A structured questionnaire was used to assess the experts’ training experience.
▪ The use of soft-embalmed cadavers was rated very positively and showed a high degree of realism. Larger sample sizes are needed to investigate a potential preference for one of the soft-embalming methods.

Tube thoracostomy (TT) training on human cadavers with soft embalming has received considerable attention in medical education and surgical skill development. Soft-embalming techniques, such as Thiel’s method, have revolutionized the use of cadavers for training purposes in various medical and surgical disciplines, including urology, plastic surgery, emergency medicine, and anesthesiology.^1^, ^2^, ^3^, ^4^ These cadavers offer advantages such as improved flexibility, color retention, and tissue properties, making them highly suitable for training in surgical and clinical procedures.^1^^,^^5^

Cadaveric training offers a unique opportunity for medical professionals to practice TT in a setting that closely mimics real-life scenarios and enabling these professionals to develop the necessary skills and confidence required for successful procedure performance.^6^ The training methods for TT have been enhanced by the integration of cadaveric simulation, which has been demonstrated to be more effective than noncadaveric simulation in achieving enhanced training outcomes because of the improved fidelity of the landmarks and tissues.^7^

The use of cadavers in training allows for a more comprehensive understanding of the anatomic variations and complexities that may be encountered during TT, ultimately leading to improved patient outcomes and safety.^7^ Overall, the use of soft-embalmed cadavers for TT training and other surgical simulations offers a promising approach for advancing medical education and skill development.

The aim of our study was to investigate the feasibility of TT on soft-embalmed human cadavers using 2 embalming methods: Thiel’s embalmed cadaver (TeC) and Dodge’s embalmed cadaver (DeC; Dodge Solutions, Dodge Co). Soft-embalming methods according to Thiel and Dodge create flexible cadavers with almost lifelike tissue conditions for surgeons, with high flexibility, clearly definable tissue layers, and good color contrasts.^8^, ^9^, ^10^ In addition, both soft-embalming types have the advantage of a long shelf life and can be stored at room temperature, unlike fresh frozen tissue, which means that TeCs and DeCs can be used multiple times, thereby reducing costs.

Although several studies have been conducted on the use of TeCs,^1^, ^2^, ^3^, ^4^ to the best of our knowledge, only 1 published study has addressed the use of DeCs in surgical workshops.^11^ Here we report on the use of DeCs for TT.

Experienced thoracic surgeons were asked to place thoracostomy tubes in a soft-embalmed cadaver. A structured questionnaire with a modified Likert scale from 1 (strongly disagree) to 5 (strongly agree) was given to surgeons to rate the closeness to reality of TT procedure on the cadavers compared with the standard performance on living patients.

## Material and Methods

This study was reviewed and approved by the local Institutional Review Board and Ethical Committee of the University of Witten (Witten, Germany; ethical approval number: S-282/2024).

### Soft-Embalmed Human Cadavers

A total of 4 soft-embalmed human cadavers were used in the current investigation: 2 TeCs8.9 and 2 DeCs.^10^

The body donations all originated in Germany and were donated to the Institute of Anatomy of the Johannes Gutenberg University Mainz (Mainz, Germany) and to the nonprofit organization MoViDo gGmbH (Essen, Germany) in accordance with the strict rules of body donation programs. The embalming of the body donations according to the Thiel method was carried out using the standard protocol at the Institute of Anatomy of the Johannes Gutenberg University Mainz. Dodge embalming was performed using the standard Dodge-protocol^10^ at the nonprofit prosecture MoViDo gGmbH. Both institutions provided the cadavers to the University of Witten/Herdecke for research and postgraduate studies.

### Study Participants

The study took place as a part of a 1.5-day workshop for video-assisted thoracoscopic surgery and included 13 specialists in thoracic surgery: a chief, 11 consultants, and 1 attending physician of thoracic surgery. All had extensive experience in TT.

### Surgical Logistics

The study was performed in the dissecting room of the Institute of Anatomy and Clinical morphology of the University of Witten/Herdecke. Regular fixed anatomy preparation tables were used, which could not be adjusted. However, the soft-embalmed cadavers were easily placed in a lateral decubitus position with both arms flexed and extended toward the head of the cadaver.

### Surgical Technique: Tube Thoracostomy Procedure

The surgeons were asked to perform a TT according to a regular procedure protocol with the cadaver in the Bülau position in the fourth intercostal space. Six surgeons performed the TT procedure on TeCs, and 7 surgeons performed the procedure on DeCs. Each participant performed the TT only once on a cadaver, either– a TeC or a DeC. The TT procedure can be divided into the 5 following steps:

1. Skin incision
2. Dissection on the upper edge of the ribs
3. Perforation of the parietal pleura
4. Insertion of the thoracic drainage
5. Suturing of the drainage

The Figure shows the drainage approach.

**Figure:**
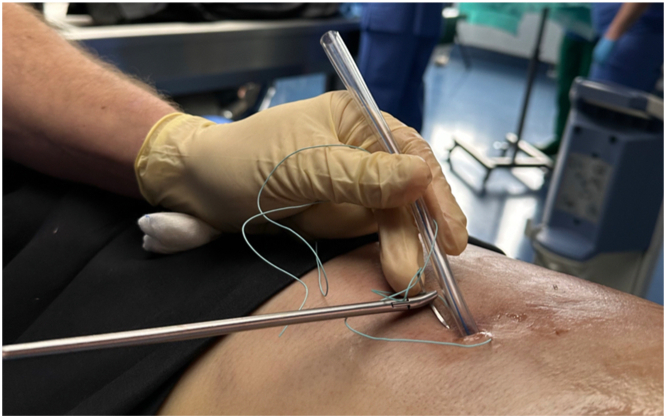
Drainage approach after tube thoracostomy showing the example of a Thiel cadaver.

### Evaluation and Statistics

A structured questionnaire was developed with 2 experts in thoracic surgery on the basis of the questionnaire model of Jaeger and colleagues.^1^ The original questionnaire is presented in the Supplemental Table.

The questionnaire, with a modified Likert scale from 1 (strongly disagree) to 5 (strongly agree), was applied to rate the closeness to reality of the TT procedure on soft-preserved cadavers compared with the standard performance on living patients. The previously mentioned 5 steps were evaluated individually in the questionnaire regarding their realism compared with the performance on a living person. In addition, 3 general questions were asked about the impression of the skills training on the soft-embalmed cadaver. The questionnaire consisted of 8 questions, and an overview can be seen in the Table.

**Table:** Overview of the Questionnaire Presented to the Experts

5 Steps of Tube Thoracostomy	
1.	The skin incision can be performed realistically.
2.	The dissection toward the upper edge of the ribs can be performed realistically.
3.	The pleural perforation can be performed realistically.
4.	The insertion of the chest drain can be performed realistically.
5.	The suturing of the drainage can be performed realistically.
General Questions	
6.	The skills training on the supplied soft-embalmed cadaver provides good preparation for performing the procedure on a live patient.
7.	I found the procedure on the soft-embalmed cadaver to be realistic.
8.	I can recommend skills training in tube thoracostomy using the soft-embalmed cadaver provided.

Descriptive analysis was used to analyze the results of the questionnaires. The analysis was carried out by determining medians with R statistical software version 4.3.1 (R Foundation for Statistical Computing).

## Results

### Procedure for Tube Thoracostomy

Regarding both methods of soft embalming, all 5 steps of the TT procedure as shown in the Table were rated with a median of 5 (strongly agree) for a realistic implementation of the procedure. For Thiel’s method, the median answer to the question about the realism of inserting the chest tube into the thorax was between 4 (agree) and 5 (strongly agree), and the remaining 4 steps of the TT procedure were rated with a median of 5 (strongly agree). For Dodge’s method, the median answer on the realism of each of the 5 steps of the TT procedure was 5 (strongly agree).

### General Questions

The question on the realism of performing TT on soft-embalmed cadavers was answered with a median of 4 (agree) for both embalming methods. Looking at the individual soft-embalming methods separately, the median answer for Thiel’s method was 4 (agree) and the median answer for Dodge’s method was 5 (strongly agree). The surgeons agreed with a median of 5 (strongly agree) for both embalming methods that the TT on soft-embalmed cadavers provides good preparation for performing the procedure on a live patient. The median answer to the question whether the experts would recommend training the TT on soft-embalmed cadavers was 5 (strongly agree).

## Comment

Chest TT is an important basic procedure in thoracic surgery, as well as in many other specialties, such as in general and trauma surgery or intensive care medicine. Realistic simulation training is an essential component of competence-based medical training, allowing trainees to practice procedures in a controlled environment without risk to patients.

In the field of emergency medicine, the rapid and accurate placement of a chest tube in the event of a tension pneumothorax has been demonstrated to be a crucial intervention with the potential to save patients’ lives in numerous instances.^12^ Furthermore, the safety and efficacy of prehospital thoracostomy performed by trained aeromedical nonphysician crews have been demonstrated, emphasizing the importance of comprehensive training in diverse settings.^12^^,^^13^ Realistic TT training can therefore lead to an improvement in the manual skills of the attending physicians. First and foremost, this can improve medical treatment for the patient.

The comparison of different training modalities for procedures such as TT has garnered significant attention, particularly regarding the effectiveness of cadaver-based training vs simulation training. Studies have compared different training modalities, that they have indicated that cadaver-based training may be more effective than simulation training for specific procedures, including TT.^6^ Tan and colleagues^14^ presented a study that compared cadaver models and simulation task trainers in teaching TT to residents. Their findings indicated that the cadaver model provided a learning experience with better procedural outcomes and higher perceived educational effectiveness compared with simulation task trainers, thus highlighting the importance of anatomic realism in cadaver training.^14^

The aim of our study was to assess the feasibility of TT on soft-embalmed human cadavers using 2 embalming methods such as those of Thiel^8^^,^^9^ and Dodge. Therefore, a structured questionnaire was used to analyze the experience reports of thoracic surgeons as experts.

Soft-embalmed TeCs^8^^,^^9^ offer advantages over traditional formalin-preserved specimens, including better preservation of tissue texture and color, which facilitates a more realistic learning environment.^15^^,^^16^ Additionally, soft-embalmed DeCs have been shown to exhibit a high degree of realism, flexibility, and color fidelity to fresh tissue.^10^^,^^11^

The results of our study according to the experts’ assessments demonstrate that TeCs and DeCs enable realistic training of TT and provide good preparation for performing the procedure on a live patient. Given the small study cohort, the evaluation results can be analyzed only descriptively and show no significant differences between TeCs and DeCs. In a direct comparison of the determined medians, a higher median value for DeCs (5; strongly agree) compared with TeCs (4; agree) can be seen in the question on the realism of performing the TT procedure. However, it remains to be seen whether significant differences between TeCs and DeCs will emerge in larger study cohorts.

In conclusion, cadavers preserved by both embalming methods, TeCs and DeCs, enable highly lifelike training for TT and were highly recommended for hands-on training in chest TT by experienced thoracic surgeons. Further studies with larger study cohorts are required for a competitive analysis with significant results using advanced statistical analysis.
