# Supplementary material for: Training of Tube Thoracostomy on Soft-Embalmed Cadavers According to Thiel and Dodge: What Do Experts Say?
Source: Ann Thorac Surg Short Rep. 2025 Jul 31;3(4):1161–5. doi: 10.1016/j.atssr.2025.07.009 (PMC12712147; doi:10.1016/j.atssr.2025.07.009)
Supplement: Supplementary Table 1 [file mmc1.pdf]

Table S1 - Questionnaire
Tube Thoracostomy

Evaluation
Please tick the appropriate box:

1. The skin incision can be performed realistically.

Strongly disagree	Disagree	Neutral	Agree	Strongly agree
1	2	3	4	5

2. The dissection towards the upper edge of the ribs can be performed realistically.

Strongly disagree	Disagree	Neutral	Agree	Strongly agree
1	2	3	4	5

3. The pleural perforation can be performed realistically.

Strongly disagree	Disagree	Neutral	Agree	Strongly agree
1	2	3	4	5

4. The insertion of the chest drain can be performed realistically.

Strongly disagree	Disagree	Neutral	Agree	Strongly agree
1	2	3	4	5

Table S1 - Questionnaire
Tube Thoracostomy

5. The suturing of the drainage can be performed realistically.

Strongly disagree	Disagree	Neutral	Agree	Strongly agree
1	2	3	4	5

6. The skills training on the supplied soft-embalmed cadaver provides good preparation for performing the procedure on a live patient.

Strongly disagree	Disagree	Neutral	Agree	Strongly agree
1	2	3	4	5

7. I found the procedure on the soft-embalmed cadaver to be realistic.

Strongly disagree	Disagree	Neutral	Agree	Strongly agree
1	2	3	4	5

8. I can recommend skills training in tube thoracostomy using the soft-embalmed cadaver provided.

Strongly disagree	Disagree	Neutral	Agree	Strongly agree
1	2	3	4	5
